# A systematic review of neuroimaging studies of adults aged 35 and older with clinical, symptomatic and genetic risk for attention-deficit/hyperactivity disorder

**DOI:** 10.1080/19585969.2026.2700984

**Published:** 2026-07-19

**Authors:** Natalia G. Docteur, Hayley G. P. Huston, Avery A. Krupa, Brandy L. Callahan

**Affiliations:** ^a^ Department of Psychology, University of Calgary, Calgary, Canada; ^b^ Hotchkiss Brain Institute, University of Calgary, Calgary, Canada

**Keywords:** Attention-deficit/hyperactivity disorder, neuroimaging, cognition, inattention, impulsivity, adults

## Abstract

Attention-deficit/hyperactivity disorder (ADHD) affects 2.5% of adults and is associated with cognitive decline and dementia. The neurobiological mechanisms contributing to adverse outcomes in ADHD are poorly understood. ADHD-related brain alterations may persist into later life and interact with ageing-related processes, potentially increasing susceptibility to neuropathology. This preregistered systematic review synthesised neuroimaging findings in adults aged 35 years and older with clinical, symptomatic, or genetic risk for ADHD, and summarised cognitive and clinical correlates. A search of five databases produced 13 included studies. Risk of bias was assessed using the Newcastle-Ottawa Scale. Most studies (11/13) had low risk of bias. Compared to controls, ADHD groups exhibited alterations in fronto-striatal, fronto-parietal, and limbic systems implicated in executive control and attention. Middle-aged adults with clinical ADHD showed more widespread cortical structural differences, whereas older adults demonstrated abnormalities primarily in frontal regions, possibly reflecting attenuation of differences through ageing. Two functional studies in those with clinical ADHD reported frontal hypoactivation alongside parietal hyperactivation, consistent with compensatory recruitment. Among undiagnosed samples, there were interactions between genetic risk for ADHD and Alzheimer’s disease-related pathology affecting brain and cognitive outcomes. Overall, ADHD-associated neurobiological alterations appear to persist into older age. Longitudinal investigations are needed to clarify these relationships.

## Introduction

Attention-deficit/hyperactivity disorder (ADHD) affects approximately 2.5% of adults globally (Song et al. 2021). While often considered a disorder of childhood, ADHD persists into adulthood in most cases, and approximately one in four individuals with the diagnosis is aged 40 or older (Kooij et al. 2019). ADHD is associated with poorer ageing outcomes including relatively limited socioeconomic achievement (Das et al. 2012), heightened depression and anxiety (Chen et al. 2018), and reduced cardio-metabolic health (Chen et al. 2018; Du Rietz et al. 2021; Li et al. 2023) compared to neurotypical peers. Notably, epidemiological research also indicates an approximate two- to six-fold increased risk of age-related neuropathological outcomes like mild cognitive impairment (MCI) and dementia (Becker et al. 2023). Although ADHD is cognitively heterogeneous (Klein et al. 2019; Callahan et al. 2021), deficits in attentional control, response inhibition, working memory, and processing speed are common (Semeijn et al. 2015; Thorell et al. 2017) and nearly one third of older adults demonstrate multi-domain cognitive impairments (Thorell et al. 2017). ADHD remains an independent risk factor for dementia beyond established predictors of neurodegenerative disease (Levine et al. 2023), indicating potential interaction with brain ageing processes to augment dementia risk (Zhang et al. 2022). However, as ADHD beyond middle age is historically understudied (Fischer and Nilsen 2024), the nature of its relationship with age-related neuropathological outcomes is not well understood.

In the past decade, four high-quality reviews have synthesised adult-inclusive neuroimaging findings in ADHD. A mega-analysis of 23 studies (Hoogman et al. 2017) reported smaller subcortical volumes (accumbens, amygdala, caudate, hippocampus and putamen) relative to controls, although the samples spanned broad age ranges (4–63 years; median age 14). Yu et al. (2022) meta-analysed 11 voxel-based morphometry (VBM) and 14 functional magnetic resonance imaging (fMRI) studies in adults, identifying diminished grey matter volumes across fronto-parietal and limbic regions, alongside mixed functional alterations with hypoactivation (insula, striatum, lenticular nucleus, superior temporal gyrus, pre- and post-central gyri, caudate nucleus, anterior thalamic projections, and the corpus callosum) and hyperactivation (middle occipital gyrus and middle temporal gyrus). ADHD participants in these studies had mean ages between 28 and 33 years (Yu et al. 2022). A review of VBM findings (Chen et al. 2025) restricted to 11 adult studies similarly showed decreased grey matter in the right superior temporal gyrus and bilateral striatum, with mean ages ranging from 20 to 66 years and substantial overlap with studies in Yu et al. (2022). Finally, a diffusion tensor imaging meta-analysis (Parlatini et al. 2023) found reduced white matter integrity in the corpus callosum and anterior thalamic radiations in ADHD, though only 25 of 129 studies examined adults exclusively.

There are currently no systematic reviews that synthesise research from relatively older ADHD samples. Our knowledge of neurodevelopmental trajectories beyond midlife is incomplete because previous studies included few older participants or treated a broad range of ages as a single group in their analyses. Thus, questions remain on whether neuropathology persists into ADHD past young adulthood and associated impacts on cognitive ageing and symptomatic expression. Improved knowledge on these topics is important for understanding mechanisms of vulnerability to age-related neuropathological outcomes.

There are several reasons why neurobiological abnormalities in ADHD may persist or change into later adulthood. Delays in brain maturation (Albajara Saenz et al. 2019; Samea et al. 2019) and altered structural and functional integrity in early life (Frodl and Skokauskas 2012; Chen et al. 2016; Yu et al. 2022) may result in subsequent compensatory mechanisms that augment later brain ageing trajectories. For instance, increased functional activation is found in frontal and parietal regions in adults with ADHD during executive function tasks, which potentially reflects alternative resource recruitment (Biehl et al. 2016). Additionally, protective effects of ADHD are also possible, as two separate cohort studies found delayed age-related decreases in subcortical volumes in adult participants (Hoogman et al. 2017; Cupertino et al. 2020), indicating selective resilience to expected brain ageing processes. Finally, ADHD is associated with lifestyle factors (Bernacer et al. 2025) and medical comorbidities (Chen et al. 2018; Du Rietz et al. 2021; Li et al. 2023) that are known determinants of accelerated brain ageing (Livingston et al. 2024). Thus, the potential additive or interactive effects of accumulated brain health risk and ADHD brain alterations may create unique susceptibility to both typical and neuropathological ageing processes, resulting in increased dementia risk.

This systematic review summarises and synthesises evidence on ADHD-associated neurobiological characteristics that may inform explanations for adverse cognitive ageing. We offer three novel contributions. First, we focus exclusively on middle age (aged 35 to 60) and older adults (older than 60) with ADHD, enabling identification of age-related patterns that may emerge after midlife. This age cut-off was selected based on converging neuroimaging and biomarker evidence indicating that key neuropathological processes emerge in the fourth decade of life, with increasing vulnerability of neural systems to metabolic and vascular stressors that precede cognitive decline (Dohm-Hansen et al. 2024; Antal et al. 2025). Second, we describe cognitive and clinical correlates of neuroimaging findings, where reported, to link brain differences with phenotypic manifestations across ageing. Insights here will bridge neurobiological findings with their implications for everyday functioning. Third, we examine neuroimaging correlates of ADHD genetic risk, recognising emerging evidence that genetic susceptibility may influence brain ageing beyond categorical clinical diagnosis (Ronald et al. 2021).

## Materials and methods

This study was conducted and reported in line with the Preferred Reporting Items for Systematic Reviews and Meta-analyses (PRISMA) 2020 Guidelines (Page et al. 2021). Prior to running the search, the review was preregistered at the Open Science Framework (https://osf.io/85efg/). The review protocol and template data collection form are publicly available.

### Search strategy

Relevant works were identified using pre-defined search terms from text, key and index words (Supplement 1). The following databases were searched: Ovid, PubMed, Web of Science, Scopus, and ProQuest Dissertation and Theses Global. A variety of databases spanning health and social sciences and including published and unpublished works were chosen to ensure broad capture of potentially relevant records. Grey literature and pre-prints were included. There were no limitations on publication date. All databases were searched on July 1, 2025.

### Eligibility criteria & definitions

All studies were deemed eligible based on the following criteria: (i) original research using primary and/or secondary data; (ii) includes only human subjects aged ≥35 years old; (iii) relevant ADHD sample based on confirmed objective clinical diagnosis, symptomatic quantification of ADHD based on objective assessment, or information regarding ADHD genetic risk; (iv) reports structural and/or functional neuroimaging findings; (v) available in English or French language.

### Study selection

Identified articles were imported into the Covidence software platform for review. Duplicates were removed automatically by the software and manually by reviewers. The first three authors independently screened titles and abstracts for exclusion in the screening stage. Each record was screened by at least two authors who were blinded to other raters’ judgments. At the full-text review stage, the same three authors assessed manuscripts against the full inclusion criteria. Each record was assessed independently by at least two authors and reasons for exclusion were recorded. Disagreements at both the screening and full-text review stage were settled by the third author. In cases where full inclusion criteria could not be confirmed, corresponding authors were contacted to gather missing information.

### Data extraction & synthesis

From the final included records, relevant data were extracted and classified into a standardised form. The first three authors extracted and verified relevant data. One author performed initial data extraction, and a second author verified extracted data for accuracy and completeness. Disagreements were settled by the third author. Extracted information included: aims, clinical and control group characteristics, sample inclusion methodology, neuroimaging type and measure, regions of interest, imaging findings, cognitive tools and outcomes, clinical assessments and outcomes, effect measures, and study conclusions. In cases where key data could not be confirmed, corresponding authors were contacted for missing information. Narrative synthesis was conducted due to heterogeneity in imaging techniques. Studies were organised by imaging, cognitive, or clinical outcome. In line with established guidance on narrative synthesis (Popay et al. 2006), findings were summarised and compared qualitatively based on effect direction, effect consistency, and study quality.

### Risk of bias assessment

Included studies were evaluated for risk of bias and quality of methodological reporting using the Newcastle-Ottawa Scale (NOS) for case-control and cohort studies (Wells et al. 2011). The NOS uses criteria for group selection, group comparability, and methods for measuring an outcome or exposure. Studies were assigned a score out of nine denoting estimated risk including low (7–9), medium (4–6), and high (0–3) risk of bias. The first two authors evaluated records for risk of bias. Each record was assessed independently by the two authors and justification for ratings were recorded. Disagreements in ratings were settled through discussion.

## Results

### Study selection and characteristics

**Figure 1.**
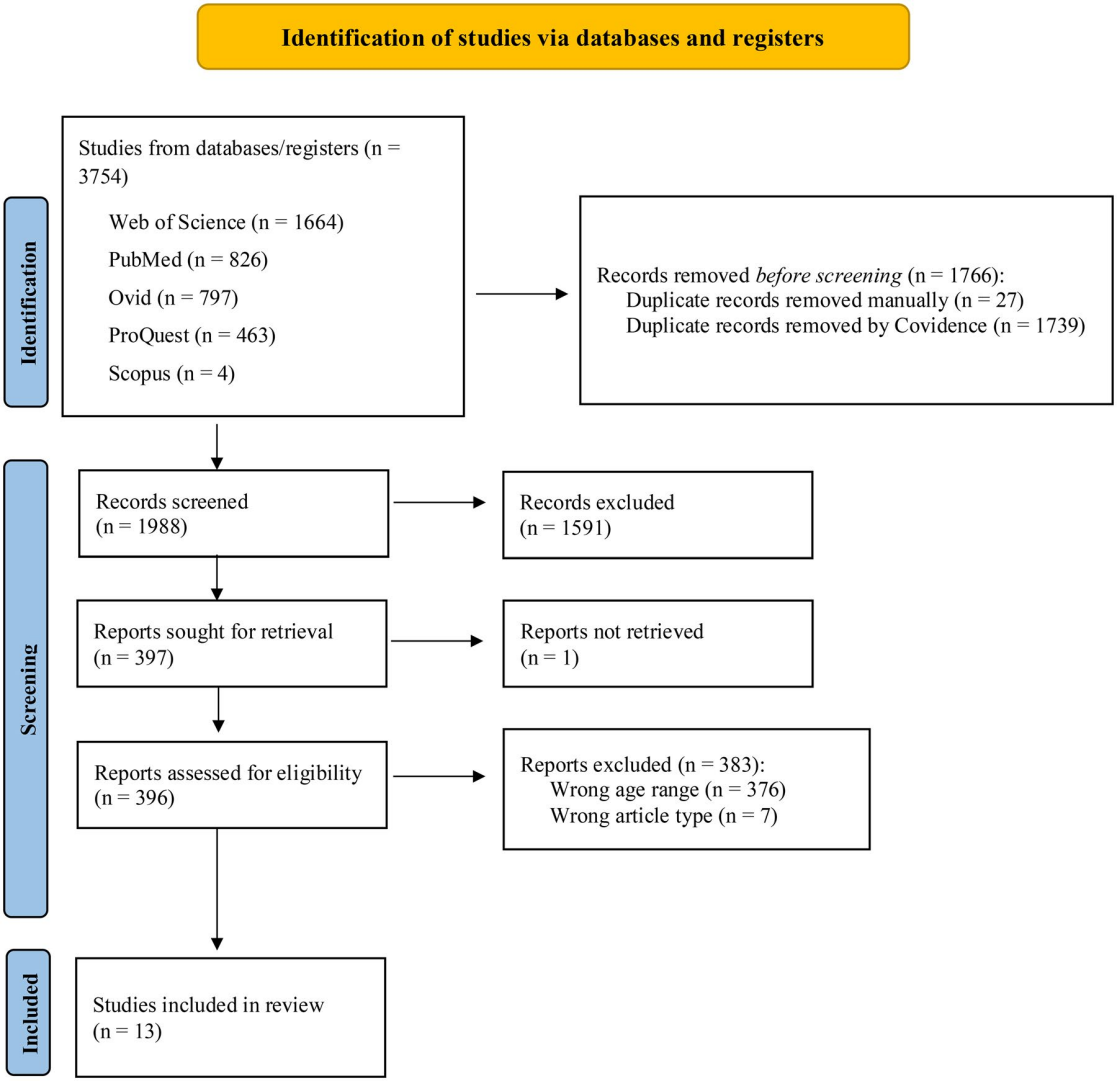
PRISMA flowchart.

In total, 13 studies were included as noted in the PRISMA flow chart (Figure 1). There was heterogeneity in operationalised definitions of ADHD, sample characteristics, data sources, and imaging techniques. Eight studies identified ADHD using clinical diagnosis with sample sizes ranging from 9 to 40 (median = 21) (Epstein et al. 2007; Amen et al. 2008; Makris et al. 2008; Proal et al. 2011; Cortese et al. 2013; Klein et al. 2021; Callahan et al. 2022; 2024). Two studies used symptomatic quantification of ADHD from population-based samples (Luders et al. 2016; Das et al. 2017) and three calculated ADHD genetic risk (Vilor-Tejedor et al. 2020; Leffa et al. 2023; 2025), with sample sizes ranging from 212 to 3,220 (median = 280). Across all included articles, mean ADHD participant ages were 41.1 to 73.5 years. Two studies (Proal et al. 2011; Cortese et al. 2013) investigated all male ADHD groups while none examined exclusively females. Six of the final included articles were primary research studies (Amen et al. 2008; Proal et al. 2011; Cortese et al. 2013; Klein et al. 2021; Callahan et al. 2022; 2024) and seven used secondary datasets (Epstein et al. 2007; Makris et al. 2008; Luders et al. 2016; Das et al. 2017; Vilor-Tejedor et al. 2020; Leffa et al. 2023; 2025). Regarding imaging methods, 10 studies utilised structural methods (Makris et al. 2008; Proal et al. 2011; Cortese et al. 2013; Luders et al. 2016; Das et al. 2017; Vilor-Tejedor et al. 2020; Klein et al. 2021; Callahan et al. 2022; Leffa et al. 2023; Callahan et al. 2024) while three used functional techniques (Epstein et al. 2007; Amen et al. 2008; Leffa et al. 2025). Three reported cognitive correlates (Epstein et al. 2007; Callahan et al. 2024; Leffa et al. 2025) and three examined clinical associations (Luders et al. 2016; Das et al. 2017; Klein et al. 2021). Main study characteristics and their results are summarised in Table 1. Synthesised findings by region/network are presented in Table 2.

**Table 1.** Main study characteristics and findings from 13 included studies.

Study	ADHD definition; data source	ADHD age M ± SD (range)	ADHD % male	ADHD sample size	Comparison group(s)	Imaging method; outcome	Imaging results	Cognitive correlates	Clinical correlates
Amen et al. (2008)	Clinically diagnosed based on DSM-IV criteria; primary data	57.1 ± 5.1 (NR; all >50)	66.7%	27	Healthy controls	SPECT; cerebral blood flow	During an executive function go/no-go task, ADHD group had lower cerebral blood flow in the bilateral orbitofrontal, precentral gyrus, premotor cortex, cerebellum and right parietal and occipital region. At rest, ADHD group had lower cerebral blood flow in left prefrontal pole and orbit, and bilateral parietal lobes and cerebellum. Effect sizes NR. Uncorrected for multiple comparisons.	N/a	N/a
Callahan et al. (2022)	Clinically diagnosed based on DSM-V criteria or ASRS; primary data	64.0 ± 8.9 (50–85)	47.5%	40	Healthy controls	MRI; cortical thickness and GMV	No GMV differences between groups. In whole-brain analyses, ADHD group had decreased cortical thickness in right pars opercularis, right lateral occipital, and right lateral orbitofrontal cortices compared to controls. In frontal lobe analyses, ADHD group had decreased cortical thickness right precentral, right pars opercularis, and bilateral lateral orbitofrontal compared to controls. Effect sizes NR. Bonferroni and cluster-wise probability corrected.	N/a	N/a
Callahan et al. (2024)	Clinically diagnosed based on DSM-V criteria; cognitively normal and MCI; primary data	63.1 ± 9.6 (48–81)	48.7%	39	Non-ADHD cognitively normal and MCI	MRI; WMH volume	ADHD group had lower periventricular WMH volumes in total (*d* = 0.67), frontal (*d* = 0.69), parietal (*d* = 0.62), and temporal (*d* = 0.45) regions. ADHD group also had lower subcortical WMH volumes in total (*d* = 0.66), frontal (*d* = 0.50), and occipital (*d* = 0.47) regions.	ADHD group showed stronger negative associations between WMH volumes and executive function. Total periventricular WMH moderated the effect of ADHD on short (*ΔR*2 = 0.06) and long-term (*ΔR*2 = 0.05) verbal memory, short-term visual memory (*ΔR*2 = 0.06), processing speed (*ΔR*2 = 0.05) and executive function (*ΔR*2 = 0.06). Total subcortical WMH moderated the effect of ADHD on short- (*ΔR*2 = 0.05) and long-term (*ΔR*2 = 0.07) verbal memory, and executive function (*ΔR*2 = 0.03). Regionally, frontal subcortical WMH moderated the effect of ADHD on long-term verbal memory (*ΔR*2 = 0.06) and executive function (*ΔR*2 = 0.04). Parietal WMH moderated the effect of ADHD on executive function (*ΔR*2 = 0.07).	N/a
Cortese et al. (2013)	Clinically diagnosed based on DSM-IV criteria; primary data	41.8 ± 3.0 (NR)	100%	15	Healthy controls, remitted ADHD	MR-DTI; white matter integrity	Compared to controls, those with ADHD in adulthood had lower white matter integrity in right sagittal stratum, external capsule, retrolenticular internal capsule, and anterior corona radiata. There were no white matter integrity differences between current and remitted ADHD in adulthood. Effect sizes NR. Corrected for multiple comparisons with threshold-free cluster enhancement.	N/a	N/a
Das et al. (2017)	Population-based sample with unknown diagnostic status; ADHD symptoms based on ASRS; secondary data from the Personality and Total Health Through Life Study	50.7 ± 1.4 (48–52)	46.8%	269	N/a	MRI; GMV	N/a	N/a	Trait inattention was positively associated with left nucleus accumbens and rostral middle frontal volumes, where final path analysis explained 28% of the variance in inattention. Trait hyperactivity was negatively correlated with left hippocampal volumes, where final path analysis explained 12% of the variance in hyperactivity. Controlling for depression and anxiety strengthened relationships between ADHD symptoms and regional volumes. Effect sizes NR. Uncorrected for multiple comparisons.
Epstein et al. (2007)	Clinically diagnosed based on DSM-IV criteria; secondary data from the Multimodal Treatment Study of ADHD	48.6 ± 9.0 (35–60)	33.3%	9	Healthy controls	fMRI; functional activation	ADHD group had lower activation in bilateral inferior frontal gyrus and left caudate, and higher activation in the anterior cingulate, precuneus and left inferior parietal lobule relative to controls. Effect sizes NR.	During an executive function go/no-go task, increased right inferior frontal gyrus activation was associated with improved target discrimination in the ADHD group (*r*=.82) compared to controls (*r*=-.15). Corrected for multiple comparisons with contiguity threshold.	N/a
Klein et al. (2021)	Clinically diagnosed based on DSM-IV criteria; primary data	66.9 ± 3.3 (NR; all ≥65)	36%	25	Healthy controls	MRI; GMV	Males with ADHD had lower total GMV compared to same-sex controls. There were no total GMV differences between females. Combining both sexes, the ADHD group had reduced GMV in right medial frontal orbital area including the medial frontal superior, frontal superior, and subgenual anterior cingulate. Effect sizes NR. Family-wise error correction for multiple comparisons.	N/a	Inattention was negatively correlated with GMV in the bilateral anterior cingulate and positively correlated with GMV in the left cerebellum. Hyperactivity/impulsivity was negatively associated with GMV in the left frontal orbital cortex. Depression was negatively correlated with bilateral caudate GMV and positively associated with right supramarginal gyrus GMV. Effect sizes NR. Family-wise error correction for multiple comparisons.
Leffa et al. (2023)	Cognitively normal with unknown ADHD diagnostic status; ADHD genetic risk based on PRS; secondary data from the Alzheimer’s Disease Neuroimaging Initiative	73.1 ± 5.9 (60–90)	45.3%	212	N/a	MRI; GMV	Higher ADHD-PRS associated with longitudinal GMV reductions over median four years in the superior frontal gyrus and supramarginal gyrus in those with high amyloid burden. No effects in those with low amyloid burden. Effect sizes NR. Random field theory to correct for multiple comparisons.	N/a	N/a
Leffa et al. (2025)	MCI and AD with unknown ADHD diagnostic status; ADHD genetic risk based on PRS; secondary data from the Alzheimer’s Disease Neuroimaging Initiative	73.5 ± 7.4 (55–91)	59.2%	938	N/a	FDG-PET; cerebral hypometabolism	In MCI, ADHD-PRS not associated with brain metabolism. In AD, higher ADHD-PRS associated with brain hypometabolism in frontal, parietal, temporal, and occipital regions and the basal ganglia. Effect sizes NR. Random field theory to correct for multiple comparisons.	In AD, parietal hypometabolism mediated the relationship between ADHD-PRS and executive function, where higher ADHD-PRS associated with decreased executive function via parietal hypometabolism. Approximately 63% of effects of ADHD-PRS on executive function was mediated by parietal lobe hypometabolism. Effect sizes NR.	N/a
Luders et al. (2016)	Population-based sample with unknown diagnostic status; ADHD symptoms based on ASRS; secondary data from the Personality and Total Health Through Life Study	70.9 ± 1.4 (69–74)	53.6%	280	N/a	MRI; cortical thickness.	N/a	N/a	In males, higher trait inattention was associated with reduced callosal thickness in the body and anterior splenium, while higher trait hyperactivity/impulsivity was correlated with lower callosal thickness in the body, anterior splenium, and border between the genu and rostrum. In females, higher trait hyperactivity/impulsivity was associated with increased callosal thickness in the posterior genu. Controlling for depression reduced the strength of correlations. Effect sizes NR. Corrected for multiple comparisons.
Makris et al. (2008)	Clinically diagnosed based on DSM-IV criteria; secondary data from the National Collaborative Perinatal project	41.3 ± 2.1 (37–46)	58.3%	12	Healthy controls	MR-DTI; white matter integrity.	ADHD group had lower white matter integrity in the right pregenual and dorsal anterior cingulum and right superior longitudinal fasciculus. There were no differences in the fornix compared to controls. Effect sizes NR. Uncorrected for multiple comparisons.	N/a	N/a
Proal et al. (2011)	Clinically diagnosed based on DSM-IV criteria; primary data	41.1 ± 2.7 (36–47)	100%	17	Healthy controls, remitted ADHD	MRI; cortical thickness and GMV	Compared to controls, those with ADHD in adulthood had lower total cortical thickness and in the bilateral frontal poles, precentral gyri, precuneus, superior temporal gyri, right middle frontal gyrus, inferior parietal lobule, and temporal gyrus extending to the insula, and left superior parietal lobule and cuneus (*d* = 0.53–0.98). Compared to controls, those with ADHD in adulthood had lower GMV in bilateral anterior cingulate, right fusiform, cuneus, and middle frontal gyrus extending to the orbitofrontal, and left superior parietal, middle temporal gyrus, temporal occipital, and inferior cerebellum (*d* = 0.56–0.96). Corrected for multiple comparisons using false discovery rate.	N/a	N/a
Vilor-Tejedor et al. (2020)	Cognitively normal with unknown ADHD diagnostic status; ADHD genetic risk based on GWAS; secondary data from The Rotterdam Study	65.3 ± 9.3 (50–100)	46.2%	3,220	N/a	MRI; GMV, WMV, ventricle volume	No significant associations with GMV or WMV in any region. Among those aged >70, genes protective against ADHD were associated with reduced longitudinal enlargement of lateral ventricles. The average longitudinal follow-up period was NR but repeat scans were conducted every 3–4 years. Bonferroni corrected for multiple comparisons.	N/a	N/a

ADHD = attention deficit/hyperactivity disorder; ASRS = Adult ADHD Self-report Scale; DSM-IV = Diagnostic and Statistical Manual of Mental Disorders, fourth edition; DSM-V = Diagnostic and Statistical Manual of Mental Disorders, fifth edition; FDG-PET = fluorodeoxyglucose positron emission tomography; fMRI = functional magnetic resonance imaging; GMV = grey matter volume; GWAS = genome wide association study; MRI = magnetic resonance imaging; MR-DTI = magnetic resonance diffusion tensor imaging; MCI = mild cognitive impairment; N/a=not applicable; NR = not reported; PRS = polygenic risk score; SPECT = single-photon emission computed tomography; WMH = white matter hyperintensity; WMV = white matter volume.

**Table 2.** Neuroimaging findings organised by network/region and imaging modality.

Network/region	Structural findings	Functional findings
Fronto-striatal	**GM:** ↓ cortical thickness/GMV in OFC, medial/lateral PFC, and ACC in adults and older adults with clinical ADHD vs controls (Proal et al. 2011; Klein et al. 2021; Callahan et al. 2022) ↔ striatal GMV in older adults with clinical ADHD vs controls (Klein et al. 2021) ADHD-PRS not associated with longitudinal striatal GMV change in cognitively unimpaired older adults (Vilor-Tejedor et al. 2020; Leffa et al. 2023) **WM:** ↓ integrity in anterior cingulum in middle-aged adults with clinical ADHD vs controls (Makris et al. 2008) ↓ integrity in anterior corona radiata and internal capsule in middle-aged adults with clinical ADHD vs controls (Cortese et al. 2013)	**fMRI:** ↑ ACC activation during inhibitory control task in middle-aged adults with clinical ADHD vs controls (Epstein et al. 2007) ↓ OFC, precentral, and premotor activation during inhibitory control task in middle-aged ADHD vs to controls (Amen et al. 2008) ↓ left caudate activation during inhibitory control task (Epstein et al. 2007); ↔ basal ganglia activation differences across tasks (Amen et al. 2008) **Metabolism:** ↑ ADHD-PRS associated with basal ganglia hypometabolism in older adults with Alzheimer’s disease and unknown ADHD diagnostic status (Leffa et al. 2025)
Fronto-parietal	**GM:** ↓ cortical thickness/GMV in superior and middle frontal gyri, pars opercularis, inferior and superior parietal lobules, supramarginal gyrus, and intraparietal regions in adults and older adults with clinical ADHD vs controls (Proal et al. 2011; Klein et al. 2021; Callahan et al. 2022) ↑ ADHD-PRS linked to longitudinal GMV loss in superior frontal and supramarginal regions in cognitively unimpaired amyloid-positive older adults (Leffa et al. 2023) **WM:** ↓ integrity in the SLF in middle-aged adults with clinical ADHD vs controls (Makris et al. 2008) ↓ frontal and parietal WMH burden in older adults with clinical ADHD vs controls (Callahan et al. 2024) WMH burden moderated ADHD-cognition relationships, including verbal memory and executive function (Callahan et al. 2024)	**fMRI:** ↓ IFG and ↑ IPL and precuneus activation during inhibitory control task in middle‑aged adults with clinical ADHD vs controls (Epstein et al. 2007) ↓ right parietal activation in similar inhibitory control paradigms in clinical ADHD relative to controls (Amen et al. 2008) ↓ IFC activation associated with better target discrimination in ADHD group only (Epstein et al. 2007) **Metabolism:** ↑ ADHD-PRS associated with hypometabolism in frontal (superior, middle frontal) and parietal (superior/inferior lobules, supramarginal gyrus, precuneus) regions in older adults with Alzheimer’s disease and unknown ADHD diagnostic status (Leffa et al. 2025) Parietal hypometabolism mediates the relationship between ADHD-PRS and executive dysfunction (Leffa et al. 2025)
Limbic and paralimbic	**GM:** ↓ cortical thickness/GMV in ACC and OFC in adults and older adults with clinical ADHD vs controls (Proal et al. 2011; Klein et al. 2021) ↔ hippocampal GMV in older adults with clinical ADHD and controls (Klein et al. 2021; Callahan et al. 2022) ↓ hippocampal GMV linked to hyperactivity/impulsivity in a middle-aged population-based sample (Das et al. 2017) ↑ nucleus accumbens GMV associated with inattention in a middle-aged population-based sample (Das et al. 2017) ↓ ACC GMV associated with inattention in clinical ADHD (Klein et al. 2021)	**fMRI:** ↑ ACC activation during inhibitory control task in middle-aged adults with clinical ADHD vs controls (Epstein et al. 2007)

*Note.* ACC = anterior cingulate cortex; ADHD = attention-deficit/hyperactivity disorder; ADHD-PRS = attention-deficit/hyperactivity disorder polygenic risk score; fMRI = functional magnetic resonance imaging; GM = grey matter; GMV = grey matter volume; IFC = inferior frontal cortex; IFG = inferior frontal gyrus; IPL = inferior parietal lobule; OFC = orbitofrontal cortex; PFC = prefrontal cortex; WM = white matter; WMH = white matter hyperintensities.; ↑ = increased; ↓ = reduced; ↔ = equal.

### Risk of bias assessment

Eleven studies were determined to have overall low risk of bias (Epstein et al. 2007; Amen et al. 2008; Makris et al. 2008; Cortese et al. 2013; Luders et al. 2016; Das et al. 2017; Vilor-Tejedor et al. 2020; Callahan et al. 2022; Leffa et al. 2023; Callahan et al. 2024; Leffa et al. 2025), while two were rated as having medium risk (Proal et al. 2011; Klein et al. 2021) (Supplement 1). The most commonly missed criteria were unexplained or unaddressed differences in response rates (e.g., minimal or no information regarding attrition, unclear handling of missing data) (Epstein et al. 2007; Amen et al. 2008; Makris et al. 2008; Proal et al. 2011; Vilor-Tejedor et al. 2020; Klein et al. 2021; Leffa et al. 2023; Callahan et al. 2024; Leffa et al. 2025), poorly defined controls (e.g., lacking comprehensive verification for absence of ADHD) (Luders et al. 2016; Das et al. 2017; Klein et al. 2021), and lacking representativeness of cases (e.g., ADHD population not captured fully due to restrictive inclusion criteria or convenience sampling) (Proal et al. 2011; Cortese et al. 2013; Klein et al. 2021).

### Imaging findings

#### Grey matter structure

Two studies investigated grey matter thickness in adults with clinically diagnosed ADHD (Proal et al. 2011; Callahan et al. 2022). Global cortical thickness was lower in middle-aged males with ADHD in adulthood relative to never-affected controls (*d* = 1.02) (Proal et al. 2011). Regional decreases were identified in 13 diffuse frontal (bilateral frontal poles and precentral gyri, right middle frontal gyrus), parietal (bilateral precuneus, left superior and right inferior lobules), and temporal areas (bilateral superior temporal gyri, right temporal gyrus extending to insula) and the left cuneus in the occipital lobe, with moderate-to-large effect sizes (*d* = 0.53–0.98). Alterations in grey matter thickness were also found in whole-brain analyses of an older sample (Callahan et al. 2022). ADHD participants had diminished cortical thickness in less cerebrally widespread but similarly located frontal regions in the right lateral orbitofrontal, right pars opercularis, and right lateral occipital cortices relative to controls (Callahan et al. 2022).

Grey matter volume was also investigated in clinically diagnosed ADHD (Proal et al. 2011; Klein et al. 2021; Callahan et al. 2022). Volumetric reductions compared to controls were present, although less robust, than the cortical thickness findings in middle-aged males (Proal et al. 2011). Those with ADHD in middle adulthood showed lower volumes in eight frontal (right middle frontal gyrus extending to orbitofrontal, bilateral anterior cingulate), parietal (left superior), and temporal areas (left middle temporal gyrus, left temporal-occipital and right fusiform) along with the right cuneus and left inferior cerebellum, with moderate-to-large effect sizes (*d* = 0.56–0.96) (Proal et al. 2011). Older ADHD samples showed somewhat aligned, albeit less striking grey matter differences. Males with ADHD had smaller total grey matter volumes compared to sex-matched controls; effects did not extend to females (Klein et al. 2021). In regional analyses combining both sexes, a cluster of reduced density in the medial frontal orbital area encompassing the medial frontal superior, frontal superior, and subgenual anterior cingulate cortex was lower than controls (Klein et al. 2021). However, all other volumetric comparisons were non-significant (Klein et al. 2021), with this predominant lack of differences across cortical and subcortical structures replicated in another older adult clinical sample (Callahan et al. 2022).

Genetic risk for ADHD predicted select volumetric changes in adults aged 60 and older (Vilor-Tejedor et al. 2020; Leffa et al. 2023). In a population-based study that did not account for diagnostic status, genes protective against ADHD were associated with less pronounced reductions in amygdala and caudate volumes, although these did not survive correction for multiple comparisons, and smaller increases in lateral ventricle size (Vilor-Tejedor et al. 2020). There were no significant associations between ADHD genetic susceptibility and total cerebral or subcortical grey matter volumes (Vilor-Tejedor et al. 2020). Among cognitively unimpaired individuals with biological risk for Alzheimer’s disease, ADHD genetic susceptibility predicted select longitudinal grey matter atrophy (Leffa et al. 2023). Increased ADHD polygenic risk was associated with larger grey matter reductions in the superior frontal gyrus and supramarginal gyrus over an average of four years among those with high amyloid burden, while there were no effects in those with low burden (Leffa et al. 2023).

#### White matter structure

Three studies reported on white matter characteristics in clinically diagnosed ADHD (Makris et al. 2008; Cortese et al. 2013; Callahan et al. 2024). Decreased white matter microstructural integrity was reported in two diffusion tensor imaging studies involving middle-aged samples (Makris et al. 2008; Cortese et al. 2013). Reduced integrity was found in right-hemispheric tracts subserving fronto-striatal (anterior corona radiata (Cortese et al. 2013), pregenual and dorsal cingulum (Makris et al. 2008)), fronto-parietal (superior longitudinal fasciculus (Makris et al. 2008)), and occipito-parietal-thalamic (sagittal stratum and retrolenticular internal capsule (Cortese et al. 2013)) connections compared to controls. There was comparably reduced white matter integrity between those with both current and remitted ADHD in adulthood relative to never-affected controls (Cortese et al. 2013). In contrast, a macrostructural study showed that older patients with ADHD had decreased white matter hyperintensity volumes representing cerebrovascular burden compared to non-ADHD peers, with the largest differences in total, frontal, and parietal periventricular and subcortical regions with moderate-to-large effect sizes (*d* = 0.50–0.69) (Callahan et al. 2024). Interestingly though, their putatively preserved white matter macrostructure was not associated with a cognitive advantage, as discussed below.

#### Functional activity

Functional activity was examined in three studies with heterogeneous sample classifications and imaging techniques (Epstein et al. 2007; Amen et al. 2008; Leffa et al. 2025). Using fMRI, middle-aged adults with clinical ADHD showed hypoactivation in fronto-striatal regions (right inferior and left frontal gyri, left caudate) and hyperactivation in the left inferior parietal lobe, precuneus, and dorsal anterior cingulate during an inhibitory control task compared to controls (Epstein et al. 2007). Another study (Amen et al. 2008) used single-photon emission computed tomography (SPECT) at rest and during an inhibitory control task in clinical ADHD participants. Aligned with previous research (Epstein et al. 2007), this investigation (Amen et al. 2008) found reductions in fronto-striatal hubs (bilateral orbitofrontal, precentral gyrus, premotor cortex), and the right parietal, occipital and bilateral cerebellar regions during task engagement; however, all subcortical comparisons were nonsignificant. At rest, there were fewer differences from controls, with lower activation in the left prefrontal pole and orbit, and bilateral parietal lobes and cerebellum (Amen et al. 2008). In older adults with Alzheimer’s disease and unknown ADHD diagnostic status (Leffa et al. 2025), genetic risk for ADHD was associated with brain hypometabolism using positron emission tomography, with the largest effects in parietal (bilateral postcentral, superior parietal, right precuneus) and frontal (bilateral paracentral, precentral, superior frontal) areas, as well as temporal and occipital areas and the basal ganglia. No such effects were seen in those with MCI (Leffa et al. 2025).

### Imaging associations with cognitive function

Three studies describe neuroimaging correlates of cognition (Epstein et al. 2007; Callahan et al. 2024; Leffa et al. 2025). In clinical ADHD, increased right inferior frontal gyrus activation was associated with improved target discrimination in middle-aged adults during an inhibitory control task (*r*=.82) while this effect did not extend to controls (*r*=-.15) (Epstein et al. 2007). Cognitive scores were similar between ADHD and non-ADHD samples, suggesting that stronger right inferior frontal gyrus recruitment was required for equivalent task performance in the ADHD group (Epstein et al. 2007). Relatedly, despite lower total and regional white matter hyperintensities, older adults with clinical ADHD had grossly similar cognition compared to controls apart from slower processing speed (*d* = 0.48) (Callahan et al. 2024). There was a moderating effect of white matter hyperintensities on the relationship between ADHD diagnosis and cognition, whereby the ADHD group suffered worse memory (Δ*R*^2^=0.05–0.07), processing speed (Δ*R*^2^=0.05), and executive function (Δ*R*^2^=0.03–0.06) for the same amount of cerebrovascular burden relative to controls, with small-to-medium effect sizes. Frontal white matter hyperintensities exacerbated the effect of ADHD on verbal memory (Δ*R*^2^=0.06) and executive function (Δ*R*^2^=0.04), while parietal white matter hyperintensities worsened the effect of ADHD on executive function (Δ*R*^2^=0.07). This indicated that clinical ADHD may increase cognitive susceptibility to pathophysiological changes acquired through ageing (Callahan et al. 2024).

Only one study examined associations between ADHD genetic risk and cognition (Leffa et al. 2025). In a sample of older adults with dementia and unknown ADHD diagnoses, there was an interactive effect of ADHD polygenic risk and Alzheimer’s disease-related pathology on cognition (Leffa et al. 2025). Higher ADHD genetic risk predicted lower executive function in patients with Alzheimer’s disease, and this effect was mediated by elevated parietal hypometabolism that accounted for 63% of the variance in cognitive performance in those with ADHD genetic susceptibility (Leffa et al. 2025). While genetic risk for ADHD was also associated with executive dysfunction in those with MCI, no regional activity measures were implicated (Leffa et al. 2025).

### Imaging associations with clinical symptoms

Three studies characterised brain structural correlates of inattention in adults with clinical and symptomatic ADHD (Luders et al. 2016; Das et al. 2017; Klein et al. 2021). Using whole-brain analyses, total grey matter volume was a poor predictor of inattention in clinical (Klein et al. 2021) and symptomatic ADHD (Das et al. 2017). In regional analyses, higher inattention was associated with reduced bilateral anterior cingulate and increased left cerebellum volumes in clinical ADHD compared to controls (Klein et al. 2021). In a population-based middle-aged sample with unknown ADHD diagnostic status, elevated inattention symptoms were significantly correlated with increased left nucleus accumbens and left rostral middle frontal volumes, and this model explained 28% of the variance in inattention (Das et al. 2017). A related study in older adults showed associations between reduced corpus callosum (body, anterior splenium) thickness and higher inattention symptoms, however this was only significant for males (Luders et al. 2016).

Regarding hyperactivity/impulsivity, total grey matter volume was not associated with this trait in either clinically diagnosed (Klein et al. 2021) or symptomatic ADHD samples (Das et al. 2017). Smaller volumes in a subregion of the inferior frontal gyrus (i.e., pars orbitalis) predicted greater hyperactivity/impulsivity in clinical ADHD (Klein et al. 2021). Within a population-based middle-aged sample with unknown ADHD diagnoses, lower left hippocampal volumes correlated with higher trait hyperactivity/impulsivity, predicting 12% of the variance (Das et al. 2017). A related study found that older males evidenced negative correlations between trait hyperactivity/impulsivity and callosal thickness (anterior splenium, body, border between the genu and rostrum), while females alternatively showed positive associations with callosal thickness in the posterior genu (Luders et al. 2016).

Neuroanatomical correlates of depression and anxiety were also reported (Luders et al. 2016; Das et al. 2017; Klein et al. 2021). Greater depression was associated with lower bilateral caudate and higher supramarginal gyrus volumes in older adults with clinical ADHD, while there were no associations with anxiety (Klein et al. 2021). However, covariance in neuroimaging outcomes between depression, anxiety and ADHD symptoms could not be determined as relationships were analysed separately (Klein et al. 2021). This question was addressed in two studies examining symptomatic ADHD (Luders et al. 2016; Das et al. 2017). Controlling for depression and anxiety strengthened associations between ADHD symptoms and regional grey matter volumes in middle-aged adults (Das et al. 2017). There were no significant direct effects of brain structure on depression or anxiety, indicating a moderating effect of concurrent psychopathology on the relationship between regional volumes and ADHD (Das et al. 2017). In an older sample, controlling for depression narrowed implicated brain regions and reduced the strength of associations between callosal thickness and ADHD symptoms in males (Luders et al. 2016). Conversely for females, adjusting for depression removed significant correlations between callosal thickness and ADHD symptoms altogether (Luders et al. 2016).

## Discussion

The present systematic review provides the first synthesis of neuroimaging findings and their phenotypic associations in adults aged 35 and older with clinical, symptomatic, and genetic risk for ADHD. Overall, results suggest that some neurobiological alterations persist into middle and older adulthood, partially converging with patterns previously identified in neuroimaging reviews including adults (Hoogman et al. 2017; Yu et al. 2022; Parlatini et al. 2023; Chen et al. 2025), while showing evidence of partial attenuation and reorganisation with age. ADHD in adulthood may overlap and interact with both typical and degenerative brain ageing processes to increase vulnerability to age-related neuropathological outcomes.

Grey matter findings in later-life ADHD suggest diminishing structural disadvantage with increasing age alongside persistent frontal vulnerability. While grey matter structure was the most frequently assessed neuroimaging outcome (Proal et al. 2011; Vilor-Tejedor et al. 2020; Klein et al. 2021; Callahan et al. 2022; Leffa et al. 2023), older adults with clinically diagnosed ADHD showed largely null differences from controls with select reductions confined to medial and lateral prefrontal regions (Klein et al. 2021; Callahan et al. 2022), whereas middle-aged males showed more widespread reductions encompassing frontal, temporal, parietal and cerebellar areas (Proal et al. 2011). Findings in middle-aged males align with prior VBM meta-analyses reporting widespread fronto-parietal and striatal grey matter reductions in adult ADHD samples (Yu et al. 2022; Chen et al. 2025). This potentially suggests age-related narrowing of affected regions. Consistent with this pattern, genetic studies in older adults without confirmed ADHD diagnoses found no association between ADHD genetic risk and longitudinal grey matter decline (Vilor-Tejedor et al. 2020), including among those with low Alzheimer’s disease biomarker burden (Leffa et al. 2023). These findings converge with evidence that ADHD-related structural deficits are more pronounced in youth (Shaw et al. 2007; Hoogman et al. 2017; 2019) than in early and middle adulthood (Nakao et al. 2011; Frodl and Skokauskas 2012; Yu et al. 2022). Persisting frontal vulnerability potentially reflects developmental lags in cortical hubs lasting into adulthood (Cortese and Castellanos 2012; Shaw et al. 2013), indicating that age may critically shape the expression of ADHD-related structural differences. However, given the small number of available studies, more research is needed to determine whether reduced grey matter differences in older adults with ADHD are truly attenuated or simply under-detected.

### Regional findings

Fronto-striatal hubs were the most consistently investigated regions, with frontal disruptions observed across adult and older adult groups (Epstein et al. 2007; Amen et al. 2008; Makris et al. 2008; Proal et al. 2011; Klein et al. 2021; Callahan et al. 2022). These findings extend literature in children, adolescents, and young adults showing delayed and deficient frontal maturation in clinical ADHD (Shaw et al. 2007; 2013; Chen et al. 2016; Hoogman et al. 2019; Cupertino et al. 2020; Yu et al. 2022), and indicate persistence beyond midlife. In neurotypical adults, frontal systems support executive functions including attention, inhibition, working memory, and motor planning (Daigle et al. 2022; Dexter and Ossmy 2023), which are among the most prominent deficits in ADHD (Semeijn et al. 2015; Thorell et al. 2017). Studies in this review linked executive performance to frontal cortical macrostructure (Callahan et al. 2024) and functional activation (Epstein et al. 2007) in clinical ADHD, suggesting continued cognitive relevance through ageing. Frontal vulnerability may contribute to executive dysfunction in later life, thereby elevating risk for dementias marked primarily by executive impairments, such as vascular and Lewy body subtypes, which are more strongly associated with ADHD than predominantly amnestic presentations like Alzheimer’s disease (Becker et al. 2023). However, small sample sizes and a scarcity of research examining frontal brain pathology and cognitive outcomes in ADHD prevent conclusive inferences on these relationships in older age.

Striatal findings were inconsistent. Across studies, both null (Amen et al. 2008; Vilor-Tejedor et al. 2020; Klein et al. 2021; Leffa et al. 2023) and significant results were reported (Epstein et al. 2007; Cortese et al. 2013; Leffa et al. 2025), perhaps attributable to methodological and design factors rather than uniform neurobiological disruption. For instance, reduced caudate activation was detected using task-based fMRI (Epstein et al. 2007), whereas a SPECT study found no basal ganglia involvement (Amen et al. 2008), potentially due to lower sensitivity for transient activations (Soares et al. 2016). Age and design differences further contextualise these findings. In undiagnosed older adults, ADHD genetic risk was unrelated to longitudinal striatal degeneration in cognitively unimpaired groups (Vilor-Tejedor et al. 2020; Leffa et al. 2023), yet predicted increased basal ganglia hypometabolism in dementia cross-sectionally (Leffa et al. 2025). Viewed alongside meta-analytic evidence of reduced striatal volumes in younger and middle-aged adults (Hoogman et al. 2017; Chen et al. 2025), and reports of slower age-related nucleus accumbens (Hoogman et al. 2017) and putamen (Hoogman et al. 2017; Cupertino et al. 2020) decline earlier in life, later-life findings suggest that subcortical abnormalities may be most pronounced earlier, with comparatively preserved structural trajectories across the adult lifespan. Although cross-sectional adult studies report reduced striatal integrity (Epstein et al. 2007; Cortese et al. 2013; Leffa et al. 2025), available longitudinal evidence in older age is more consistent with relative preservation over time. This interpretation is supported by relatively spared putamen and caudate volumes in unaffected siblings of young adults with ADHD versus youth comparisons (Greven et al. 2015), indicating stronger early genetic influences on striatal development. Mechanistically, striatal alterations may reflect atypical functional organisation related to dense dopaminergic innervation, including abnormal dopamine receptor availability and compensatory dopamine signalling (MacDonald et al. 2024). Future research should clarify how later-life striatal function relates to clinical features such as hyperactivity/impulsivity, which declines with age (Callahan and Plamondon 2019) and is linked to striatal abnormalities (Dang et al. 2016).

Fronto-parietal alterations in later-life ADHD were consistent with disrupted or compensatory control network functioning. Although findings were mixed, clinically diagnosed adults tended to show parietal vulnerability in late life. Structurally, middle-aged samples showed reduced parietal integrity (Makris et al. 2008; Proal et al. 2011), while in older adults parietal cerebrovascular burden was associated with worse executive function (Callahan et al. 2024). Given the established role of fronto-parietal networks in attentional control and cognitive flexibility (Rubia 2018; Dexter and Ossmy 2023), persistent ADHD-related abnormalities in older age may contribute to relatively poorer neuropathological outcomes. Functionally, atypical parietal recruitment may reflect compensatory engagement of control networks in ADHD. Studies reported right hemispheric hypoactivation (Amen et al. 2008) and left hemispheric hyperactivation (Epstein et al. 2007) during sustained attention and inhibitory control tasks in ADHD, with increased parietal activation occurring alongside comparable task performance relative to controls (Epstein et al. 2007). This follows from young adults (Biehl et al. 2016), where bilateral task-related parietal recruitment has been interpreted as compensatory or alternative recruitment, and extends fMRI meta-analytic reports of mixed frontal-parietal hypo- and hyperactivation in those with ADHD (Yu et al. 2022), indicating that heterogeneity and compensatory dynamics persist into later life rather than resolving with age. Atypical parietal recruitment is also linked to frontal deactivations in attentionally-demanding contexts (Rubia 2018), with models proposing that prefrontal and anterior cingulate impairments in ADHD constrain higher-order coordination to produce more effortful responding (Fassbender and Schweitzer 2006). While bilateral parietal recruitment is often interpreted as an adaptive response to declining network integrity in neurotypical ageing (Kang et al. 2021), ADHD may involve earlier or more pronounced reliance on similar mechanisms. Longitudinal functional investigations will be critical for clarifying the emergence, stability, and cognitive consequences of alternative recruitment in later-life ADHD.

Limbic and paralimbic alterations in ADHD appear symptom-specific rather than reflecting diffuse structural abnormalities (Luders et al. 2016; Das et al. 2017; Klein et al. 2021). In clinically diagnosed older adults, inattention was linked to smaller anterior cingulate volumes (Klein et al. 2021), suggesting that limbic-prefrontal dysregulation may underlie impaired attention and conflict monitoring, perhaps due to the anterior cingulate’s role in integrating emotional salience with cognitive control (Bledsoe et al. 2013). In population-based middle-aged cohorts, trait hyperactivity/impulsivity was associated with reduced left hippocampal volumes, potentially reflecting impaired inhibitory control (Das et al. 2017). Stress-related mechanisms may contextualise hippocampal findings in ADHD, given links between hyperactivity/impulsivity and biological stress markers in clinical ADHD (Saccaro et al. 2021), and between stress exposure (Lindgren et al. 2016; Zimmerman et al. 2016) and reduced hippocampal volumes in non-ADHD groups. Although hippocampal differences were unremarkable in two older adult samples (Klein et al. 2021; Callahan et al. 2022), future research should examine how symptom profiles and stress exposure interact, particularly given the adverse effects of stress on cognition (D’Amico et al. 2020). Affective comorbidity also may shape limbic-symptom relationships. Depression and anxiety modified associations between ADHD symptoms and regional brain volumes, with several relationships attenuated after accounting for affective symptoms (Luders et al. 2016; Das et al. 2017). Accordingly, future studies should explicitly model depression and anxiety to address their potential confounding influence. More broadly, age-related vulnerability of limbic systems (Raz et al. 2010) may interact with ADHD-related neurobiological differences (Das et al. 2017; Klein et al. 2021) and affective comorbidities, amplifying ADHD symptom expression through ageing.

### Potential interactive links between ADHD and neuropathological outcomes

The extent to which brain alterations in ADHD contribute to age-related neuropathological outcomes is unclear. However, emerging research linking ADHD-associated genes with Alzheimer’s disease biomarkers (Leffa et al. 2023) and clinical diagnoses (Leffa et al. 2025) suggests possible interactive effects. In cognitively unimpaired older adults with unknown ADHD diagnoses, higher ADHD polygenic risk was associated with reduced supramarginal and superior frontal gyrus volumes only in the presence of elevated amyloid burden (Leffa et al. 2023). Similarly, among patients with clinical Alzheimer’s disease, higher ADHD genetic risk was linked to poorer executive function (Leffa et al. 2025), mediated by increased parietal hypometabolism. These findings indicate that ADHD genetic liability may confer vulnerability in the context of Alzheimer’s neuropathology, potentially reflecting convergence on partially shared fronto-parietal circuits implicated in early degenerative processes (Jacobs et al. 2012) and identified as altered in several studies reviewed here (Amen et al. 2008; Makris et al. 2008; Proal et al. 2011; Leffa et al. 2023; 2025). From this perspective, ADHD-related neurobiological differences may predispose some individuals to greater susceptibility to poor brain ageing; however, further research is needed to replicate and expand on these findings.

The cumulative effects of lifestyle, vascular, and metabolic risk may further increase susceptibility to age-related neuropathological outcomes in later-life ADHD. Individuals with clinically diagnosed ADHD have higher rates of adverse lifestyle factors (e.g., smoking, problematic alcohol use, and sedentary behaviours) (Bernacer et al. 2025) and cardio-metabolic disease (e.g., stroke, diabetes, and obesity) (Chen et al. 2018; Du Rietz et al. 2021; Li et al. 2023) that contribute to neurodegenerative pathways linked to cognitive decline and dementia (Livingston et al. 2024). One study identified in this review found that although older adults with ADHD exhibited lower overall white matter hyperintensities, they experienced greater impairments in memory, processing speed, and executive function at higher levels of cerebrovascular burden (Callahan et al. 2024), suggesting heightened cognitive vulnerability to equivalent vascular injury. While mechanisms underlying this putative sensitivity remain unclear, ADHD genetic liability may also be linked to vascular processes such as atrial fibrillation (Vilor-Tejedor et al. 2020), an established risk factor for accelerated cognitive decline (Smith et al. 2024). These findings highlight potential interactions between ADHD, genetic risk, and vascular and lifestyle factors that penalise brain health in later life. Further research on shared mechanisms between ADHD and dementia risk is warranted to clarify these relationships.

### Limitations and future directions

Several limitations should be considered when interpreting findings from this review. Substantial heterogeneity in imaging methods and ADHD classification approaches restricted inferences regarding neurobiological ageing trajectories and precluded meta-analytic synthesis. Future work using harmonised imaging protocols and consistent diagnostic definitions will be essential for clarifying age-related patterns. Most included studies had small sample sizes, and only two conducted sex-stratified analyses (Luders et al. 2016; Klein et al. 2021), limiting conclusions about potential sex differences in later-life neuroimaging characteristics. Given evidence for sex-specific neurobiological differences in younger ADHD populations (Carucci et al. 2023), future work should examine whether similar patterns persist in older adults, which may inform sex-specific or life-stage-sensitive interventions. For instance, oestrogen loss during menopause influences dopaminergic modulation in neurotypical females, with downstream impacts on cognition and mood (Del Río et al. 2018). Because ADHD is associated with atypical dopamine signalling (MacDonald et al. 2024), females may be particularly neurobiologically vulnerable to age-related hormonal changes, highlighting the peri- and post-menopausal stages as potentially important windows for targeted monitoring and intervention. Further, medication use was inconsistently reported across studies, restricting evaluation of its potential confounding effects. Although psychostimulant treatment is not strongly associated with structural brain differences in ADHD (Hoogman et al. 2017), it may influence functional networks (Albajara Saenz et al. 2019); future studies should more systematically measure and model medication exposure. Finally, all studies examining clinical and symptomatic ADHD were cross-sectional, requiring age-related trajectories to be inferred from between-group comparisons. Longitudinal investigations are critically needed to characterise individual ageing trajectories and interindividual variability, particularly given opposing age-related trends in inattentive and hyperactivity/impulsivity symptoms (Kooij et al. 2019).

This systematic review also has limitations. Included studies were not uniformly high quality, limiting the robustness of conclusions we are able to draw. The small number of available studies reflects the scarcity of neuroimaging research in ADHD beyond midlife, and most samples were predominantly White, raising uncertainty about the generalisability of findings to more diverse populations ageing with ADHD. Finally, the use of multiple ADHD classification approaches introduces heterogeneity in conceptualisation and comparability, warranting caution when drawing overarching conclusions across populations.

As the clinical needs of adults ageing with ADHD become increasingly apparent, greater clarity regarding neurobiological ageing in this population is needed. Taken together, findings from this review refine conclusions from prior adult neuroimaging syntheses by suggesting that while core fronto‑striatal and fronto‑parietal vulnerabilities persist into later life, their structural and functional expression may attenuate, reorganise, or interact with ageing‑related processes. Longitudinal and methodologically harmonised research will be essential for defining brain ageing trajectories in ADHD and their clinical relevance.

## Supplementary Material

Supplemental Material
