# Supplementary material for: A systematic review of neuroimaging studies of adults aged 35 and older with clinical, symptomatic and genetic risk for attention-deficit/hyperactivity disorder
Source: Dialogues Clin Neurosci. 2026 Jul 19;28(1):259–76. doi: 10.1080/19585969.2026.2700984 (PMC13470905; doi:10.1080/19585969.2026.2700984)
Supplement: Supplementary file 1 [file TDCN_A_2700984_SM9958.docx]

Table of Contents

[Search terms for each database	2](#_Toc220596088)

[PRISMA flow chart details	4](#_Toc220596089)

[Newcastle-Ottawa Scale (NOS) scores for 13 included studies	6](#_Toc220596090)

# Search terms for each database

**OVID**

Search terms:

((adult OR adults OR elderly OR geriatric) AND (ADHD OR Attention-deficit hyperactivity disorder OR attention deficit hyperactivity disorder OR attention deficit disorder) AND (Neuroimaging or Imaging OR MRI OR fMRI OR Magnetic resonance OR Positron emission tomography OR Positron tomography OR PET OR VBM OR Voxel-based OR Morphometry OR DTI OR Diffusion tensor imaging OR Microstructure OR Macrostructure OR Microstructural OR Gray matter OR Grey matter OR White matter OR Morphology OR Brain networks)).ab,ti.

**PUBMED**

Search terms:

((adult[Title/Abstract] OR adults[Title/Abstract] OR elderly[Title/Abstract] OR geriatric[Title/Abstract]) AND (ADHD[Title/Abstract] OR Attention-deficit/hyperactivity disorder[Title/Abstract] OR attention deficit hyperactivity disorder[Title/Abstract] OR attention deficit disorder[Title/Abstract])) AND (Neuroimaging[Title/Abstract] OR Imaging[Title/Abstract] OR MRI[Title/Abstract] OR fMRI[Title/Abstract] OR Magnetic resonance[Title/Abstract] OR Positron emission tomography[Title/Abstract] OR Positron tomography[Title/Abstract] OR PET[Title/Abstract] OR VBM[Title/Abstract] OR Voxel-based[Title/Abstract] OR Morphometry[Title/Abstract] OR DTI[Title/Abstract] OR Diffusion tensor imaging[Title/Abstract] OR Microstructure[Title/Abstract] OR Macrostructure[Title/Abstract] OR Microstructural[Title/Abstract] OR Gray matter[Title/Abstract] OR Grey matter[Title/Abstract] OR White matter[Title/Abstract] OR Morphology[Title/Abstract] OR Brain networks[Title/Abstract])

**WEB OF SCIENCE**

Search terms:

((TS=(adult or adults or elderly or geriatric)) AND TS=(ADHD or Attention-deficit/hyperactivity disorder or attention deficit hyperactivity disorder or attention deficit disorder)) AND TS=(Neuroimaging OR Imaging OR MRI OR fMRI OR Magnetic resonance OR Positron emission tomography OR Positron tomography OR PET OR VBM OR Voxel-based OR Morphometry OR DTI OR Diffusion tensor imaging OR Microstructure OR Macrostructure OR Microstructural OR Gray matter OR Grey matter OR White matter OR Morphology OR Brain networks)

**SCOPUS**

Search terms:

(TITLE-ABS-KEY (adult OR adults OR elderly OR geriatric) AND TITLE-ABS-KEY (ADHD OR Attention-deficit / hyperactivity disorder OR attention deficit hyperactivity disorder OR attention deficit disorder) AND TITLE-ABS-KEY (Neuroimaging OR Imaging OR MRI OR fMRI OR Magnetic resonance OR Positron emission tomography OR Positron tomography OR PET OR VBM OR Voxel-based OR Morphometry OR DTI OR Diffusion tensor imaging OR Microstructure OR Macrostructure OR Microstructural OR Gray matter OR Grey matter OR White matter OR Morphology OR Brain networks))

**PROQUEST**

Search terms:

Summary(adult OR adults OR elderly OR geriatric) AND summary(ADHD OR Attention-deficit/hyperactivity disorder OR attention deficit hyperactivity disorder OR attention deficit disorder) AND summary(Neuroimaging OR Imaging OR MRI OR fMRI OR Magnetic resonance OR Positron emission tomography OR Positron tomography OR PET OR VBM OR Voxel-based OR Morphometry OR DTI OR Diffusion tensor imaging OR Microstructure OR Macrostructure OR Microstructural OR Gray matter OR Grey matter OR White matter OR Morphology OR Brain networks)

# PRISMA flow chart details

Of the 383 studies assessed for eligibility and ultimately excluded at full-text screening:

98.17% (n = 376) were excluded based on wrong age range of participants, including but not limited to the below studies with clinical/symptomatic/genetic ADHD participants <35 years of age.

- Halbe, E., Jamieson, A., Bergmann, M., Mehren, A., Harrison, B. J., Davey, C. G., ... & Lux, S. (2025). Neural Correlates and Sex‐Specific Effects of Affectively Driven Processes Underlying Decision‐Making in Adult ADHD. *Brain and behavior*, *15*(3), e70215.
- Blomberg, R., Signoret, C., Danielsson, H., Perini, I., Rönnberg, J., & Capusan, A. J. (2022). Aberrant resting-state connectivity of auditory, ventral attention/salience and default-mode networks in adults with attention deficit hyperactivity disorder. *Frontiers in Neuroscience*, *16*, 972730.
- Berberat, J., Huggenberger, R., Montali, M., Gruber, P., Pircher, A., Lövblad, K. O., ... & Remonda, L. (2021). Brain activation patterns in medicated versus medication-naïve adults with attention-deficit hyperactivity disorder during fMRI tasks of motor inhibition and cognitive switching. *BMC Medical Imaging*, *21*(1), 53.
- Bandeira, C. E., Grevet, E. H., Vitola, E. S., da Silva, B. S., Cupertino, R. B., Picon, F. A., Ito, L. T., Tavares, M. E. A., Rovaris, D. L., Grimm, O., & Bau, C. H. D. (2024). Exploring Neuroimaging Association Scores in adulthood ADHD and middle-age trajectories. Journal of psychiatric research, 176, 348–353.
- Heinrichs-Graham, E., Franzen, J. D., Knott, N. L., White, M. L., Wetzel, M. W., & Wilson, T. W. (2014). Pharmaco-MEG evidence for attention related hyper-connectivity between auditory and prefrontal cortices in ADHD. Psychiatry research, 221(3), 240–245. https://doi.org/10.1016/j.pscychresns.2014.01.002

1.83% (n = 7) were excluded based on being the wrong article type (e.g., abstracts, meta-analyses, reviews):

- Ivanov, I., Newcorn, J. H., Krone, B., Li, X., Duhoux, S., White, S. F., ... & Blair, R. J. (2021). Neurobiological Basis of Reinforcement-Based Decision-Making in Adults With ADHD Treated With Lisdexamfetamine Dimesylate. *Journal of Attention Disorders*, *25*(11), 1632-1633.
- Xie, H., Cao, Y., Long, X., Xiao, H., Wang, X., Qiu, C., & Jia, Z. (2022). A comparative study of gray matter volumetric alterations in adults with attention deficit hyperactivity disorder and bipolar disorder type I. *Journal of Psychiatric Research*, *155*, 410-419.
- Nikolaus, S., Mamlins, E., Giesel, F. L., Schmitt, D., & Müller, H. W. (2022). Monoaminergic hypo-or hyperfunction in adolescent and adult attention-deficit hyperactivity disorder?. *Reviews in the Neurosciences*, *33*(4), 347-364.
- Paloyelis, Y., & Asherson, P. J. (2011). Structural and functional magnetic resonance imaging findings in adults with ADHD. *ADHD in adults*, 49-65.
- Ahn, K., Norman, L. J., Justice, C. M., & Shaw, P. ADHD and its neurocognitive substrates: a two sample mendelian randomization study. *Transl Psychiatry. 2022; 12: 378*.
- Schulze, M., Aslan, B., Lux, S., & Philipsen, A. (2022). Deficient Multisensory Integration with concomitant resting-state connectivity in adult ADHD. *European Psychiatry*, *65*(S1), S332-S332.
- Aron, A. R. (2003). *The neural basis of inhibitory mechanisms in executive function* (Doctoral dissertation, University of Cambridge).

# Newcastle-Ottawa Scale (NOS) scores for 13 included studies

| **Study** | **Selection** | | | | **Comparability** | **Exposure** | | | **Total Score** |
| --- | --- | --- | --- | --- | --- | --- | --- | --- | --- |
|  | Criteria 1 (0 or 1) | Criteria 2 (0 or 1) | Criteria 3 (0 or 1) | Criteria 4 (0 or 1) | Criteria 1 (0 or 1 or 2) | Criteria 1 (0 or 1) | Criteria 2 (0 or 1) | Criteria 3 (0 or 1) |  |
| Amen et al. (2008) | 1 | 1 | 1 | 1 | 1 | 1 | 1 | 0 | 7 |
| Callahan et al. (2022) | 1 | 1 | 0 | 1 | 2 | 1 | 1 | 1 | 8 |
| Callahan et al. (2024) | 1 | 1 | 0 | 1 | 2 | 1 | 1 | 0 | 7 |
| Cortese et al. (2013) | 1 | 0 | 1 | 1 | 2 | 1 | 0 | 1 | 7 |
| Das et al. (2017) | 1 | 1 | 1 | 0 | 2 | 1 | 1 | 1 | 8 |
| Epstein et al. (2007) | 1 | 1 | 1 | 1 | 2 | 1 | 1 | 0 | 8 |
| Klein et al. (2021) | 1 | 0 | 1 | 0 | 2 | 1 | 1 | 0 | 6 |
| Leffa et al. (2023) | 1 | 1 | 1 | 1 | 2 | 1 | 1 | 0 | 8 |
| Leffa et al. (2025) | 1 | 1 | 1 | 1 | 2 | 1 | 1 | 0 | 8 |
| Luders et al. (2016) | 1 | 1 | 1 | 0 | 2 | 1 | 1 | 1 | 8 |
| Makris et al. (2008) | 1 | 1 | 1 | 1 | 2 | 1 | 1 | 0 | 8 |
| Proal et al. (2011) | 1 | 0 | 1 | 1 | 1 | 1 | 0 | 0 | 5 |
| Vilor-Tejedor et al. (2020) | 1 | 1 | 1 | 1 | 2 | 1 | 1 | 0 | 8 |

*Note.* Scores represent consensus ratings after discussion.

NOS scoring was completed in accordance with published criteria: <https://www.ncbi.nlm.nih.gov/books/NBK261415/>
